# Health management students’ self-regulation and digital concept mapping in online learning environments

**DOI:** 10.1186/s12909-021-02542-w

**Published:** 2021-02-17

**Authors:** Dorit Alt, Lior Naamati-Schneider

**Affiliations:** 1 Kinneret College on the Sea of Galilee, Tzemach Junction, 15132 Jordan Valley, MP Israel; 2 Hadassah Academic College, 37 Hanevi’im St., 9101001 Jerusalem, Israel

**Keywords:** Health management education, Self-regulation of learning, Digital concept mapping, Problem-based learning

## Abstract

**Background:**

Self-regulation of learning is considered one of the key capabilities deemed essential for the healthcare system and its workers to cope successfully with the current challenges they are facing. Therefore, healthcare curricula are increasingly called upon to support self-regulation as a central learning outcome. With scant relevant publications describing how students of medicine and other healthcare professions regulate their learning, this study set out to design and assess a problem-based learning using digital concept mapping in an online course and to evaluate the set of connections between this intervention and Health Management students’ self-regulation of learning.

**Method:**

Students of a Management of Health Service Organizations program (100) were presented with an ill-structured problem, relevant to their course content (accreditation process within hospitals) and were asked to argue for or against the implementation of the accreditation process. The participants were asked to detail five arguments to establish their decision by using *Mindomo*, a popular digital platform for designing concept maps. The students were given predefined criteria that allowed them to self-assess their maps. Data for the analysis were gathered by two measurements: Concept mapping for problem-based learning scale and the Online self-regulated learning scale and were analyzed by using Partial Least Squares - Structural Equation Modeling.

**Results:**

The analyses showed that at the beginning of the process, students’ online self-regulation was found lower than at the end of the intervention, and only two self-regulation sub-factors: Goal setting and Task strategies, were positively linked to students’ perceptions of the intervention. After the intervention, the analyses showed that it increased the levels of four Online self-regulation sub-factors: Goal setting, Task strategies, Environment structuring, and Time management.

**Conclusions:**

Teachers need to recognize and account for different types of learners and encourage and scaffold students’ effective use of self-regulation strategies. Low self-regulated learners might fail to see the advantages of concept mapping in problem-solving activities. Combining these teaching and learning tools together with the use of advanced technology in an online course that encourages active learning enables the development and acquisition of abilities of self-directed learning among students in the medical and health management professions.

## Introduction

The twenty-first century has undoubtedly brought about numerous and profound changes, developments, and advances in complex technologies and in the control they exert over many spheres of our lives, alongside rapid accumulation of knowledge. These widespread changes have also had an impact upon the medical profession. The healthcare system and its professionals must cope with constant changes and new challenges that require adapting the system to the dynamic, ever-changing era. These challenges are linked to the ever-increasing use of advanced technology, artificial intelligence, and alterations in the nature and scope of the medical and nursing profession [1]. These trends are occurring alongside changes such as the introduction of many new regulations and reforms, the lengthening lifespan, and various demographic changes. The medical field is also facing market conditions characterized by decreasing and limited resources together with increasing competition among multiple entities, including the doctors themselves [2, 3]. Accordingly, a set of qualifications is currently required of managers and staff in the healthcare system such as flexibility, adaptability, planning ability, and self-regulation of learning (SRL). These capabilities are becoming an essential and inseparable part of the array of tasks that characterize doctors in the twenty-first century [4, 5]. Based on this notion, cultivating these critical lifelong learning thinking skills has become the focus of modern-day medical education [6–10]. The objective is to train medical managers and doctors to adapt themselves to the new era and its corresponding demands. SRL is deemed essential among these learning skills throughout life for doctors and healthcare workers. It enables flexibility and adjustment to dynamic changes in the health market that are similar throughout the world [11, 12].

This raises the call to develop curricula that can support healthcare students in developing their SRL, thereby helping them to be proactive, behaviorally, metacognitively and motivationally, in their learning process. In view of the importance of this issue and the need to assimilate the development of these abilities among medical professionals and students in the medical field, and in view of the scant research conducted in this field, the present study focuses on promoting SRL.

This study sought to design and assess a problem-based learning (PBL) intervention activity with digital concept mapping (CM) in an online course and to evaluate the set of connections between this process and Health Management students’ SRL [13–15]. More specifically, we were interested in evaluating the impact of the decision-making process required in a PBL activity on the manner in which students construe their autonomy in an online learning environment. Illuminating these associations may enhance our understanding of how to effectively use CM for PBL in online courses so as to develop lifelong learning skills for medical personnel and medical managers at an early stage of their training. These abilities will enable medical professionals to better contend with a variety of needs in the digital age and with the changes in, and dynamics of, technological and medical knowledge.

## Literature review

### Self-regulation of learning in healthcare curricula

SRL is considered a core competence of healthcare professionals and one that is essential to the safeguarding of patient care [16]. In the health professions, we expect practitioners and trainees to engage in self-regulation of their learning and practice [17]. For example, medical professionals have to ensure high standards in the provision of patient care in the context of a rapidly and constantly changing medical world. Doctors are responsible for diagnosing their own learning needs and pursuing professional development opportunities; medical residents are expected to identify their knowledge gaps and to seek help from supervisors when they need it [11]. To this end, they have to continuously develop their competencies, define their own learning needs, set personal goals, and engage in the most appropriate learning activities. It means, in essence, that they have to be self-regulated learners [18].

SRL refers to students’ ability to understand and control their learning [19], to the degree to which individuals become metacognitively, motivationally, and behaviorally active participants in their own learning processes [20]. Metacognitive control is the regulation of skills for planning, monitoring, and modifying cognition, which refers to the awareness and control of one’s thought processes. Self-regulated learners use cognitive strategies designed to increase encoding, understanding, and/or retention of learning, to pursue academic goals and regulatory strategies that enable them to self-monitor and control their own learning [21]. Motivational control is the regulation of beliefs and attitudes, self-efficacy, perceptions of task difficulty, task value beliefs, and personal interest in the task [22].

Despite the importance of SRL for students, a systematic search for relevant publications describing how undergraduate and/or postgraduate medical students regulate their learning points to merely a scant theoretical framework [3]. These reviews mainly indicate that students often struggle to regulate their learning in clinical learning environments as a result of the unpredictable, dynamic and disorganized nature of clinical workplace settings [11]. Therefore, healthcare curricula are increasingly called upon to support self-regulation as a central learning outcome. This raises the call to develop curricula that might support healthcare students in developing SRL, by designing constructivist-based learning environments, thus helping students to be behaviorally, metacognitively and motivationally proactive in their learning process. The learning environment suggested in the present study is an online PBL supported by CM. The following sections provide a comprehensive definition of this learning environment and show its increased importance as recorded in the literature for advancing SRL in health education.

### Concept maps for constructivist problem-based learning

Concept maps (CMs) [23] have been applied in education systems for more than 30 years [24]. However, their application to PBL has been less commonly utilized in higher education, and minimal attention has been paid to their use in online learning. CM is a method of learning and an educational tool using diagrammatic interrelationships between concepts representing subject knowledge. CMs should not simply list information from text randomly but rather should depict the structure of knowledge in propositional statements that illustrate the interrelationships between the concepts in a map [25].

The process enables one to organize and structure information and the interrelationships between them within a particular domain. This may be done in a wholly graphical manner (i.e., using images, photos, colors etc.) to highlight differing concepts and their linkages, or by identifying key concepts by their names or titles and enclosing them in visual boxes [26]. CMs may aid the instructor in assessing what students understand and how they relate the material to the overall course goals. CMs are easily taught and can be incorporated in introductory units, midterm reviews and assessments, peer assessment, self-assessment, or end-of-course reviews and assessments [27].

Machado and Carvalho [28] have recently reviewed the benefits and challenges of CM in higher education. Based on their findings, CM promotes critical thinking [29]; has a positive effect on exam scores [30]; allows the integration of theory with practice, and links new knowledge with existing knowledge [31]; promotes learning progress and assessment [32]; enhances collaborative learning [33]; increases technology inclusion [34]; enables meaningful learning [35]; and is considered an educational tool to facilitate learning and studying [36]. For example, in a recent study Chen and Hwang (2020) showed the benefits of concept mapping to English as a foreign language (EFL) learners’ critical thinking awareness and learning performance [37]. In the context of health and clinical education, recent studies showed the advantages of CM to nursing students’ clinical reasoning abilities [38]; motivation for critical thinking skills [6]; critical thinking skills [7, 10]; active learning and connections to clinical concepts [8].

Among these multifaceted benefits, critical thinking and problem solving are identified as key skills essential for learners in the twenty-first century [39]. Proficiency in critical thinking is a skill required not only in higher education settings but also in the workplace and in personal, social, and civic life [40]. Students need to know how to frame, analyze, and synthesize information to solve problems and answer questions critically. However, researchers [41, 42] maintain that question posing is challenging for many students, and without appropriate scaffolding it may lead to a heavy cognitive load. Their studies show how concept mapping activities with designated guidance can positively affect learning achievement and question-posing performance and help guide students to learn in a more effective way.

Many studies on CM have focused on health education, arguing that in today’s challenging and highly complex healthcare settings, students must be able to think critically [43, 44]. The current traditional and rote methods of learning are inefficient in eliciting critical thinking skills among the students; therefore, educators must find a different teaching method to encourage students to make the analytical thinking process part of their daily practice. Educators need to adopt instructional strategies to equip students with knowledge in critical thinking, creative problem solving and collaboration [45].

Through CM, students should be able to transfer applied didactic objectives from the classroom to their clinical practice, where critical thinking and problem-solving skills are vital for success. This premise was reinforced by several studies that empirically showed the benefits of CM used with PBL in facilitating students’ twenty-first century skills. For example, Joshi and Vyas [44] maintain that CM should be used in PBL to solve epidemiological problems in community medicine which deals with public health concepts, mathematical calculations, and “applied” interpretations. In their study, they compared two groups of students: the research group was informed on how to make and use CMs out of taught contents, while the control group was taught the same contents in the conventional way. Performance of both groups was assessed by two identical exams. The study group consistently scored higher on the exams and provided largely positive feedback on the utility of CMs in memorizing, confidence-boosting, and understanding the topic. Similarly, CM’s effectiveness in academic performance in problem-solving as well as in declarative knowledge questions and their perceptions regarding CM was examined among medical students [46]. These researchers found that CM improved academic performance in problem solving but not in declarative knowledge questions. Students’ perception of the effectiveness of CM was positive.

A qualitative study with 20 undergraduate nursing students sought to identify whether the use of CM can help students extend and revise their expertise in oncology and analyze the abilities they developed in order to convert theoretical knowledge into practical knowledge [44]. The resource of graphics and the clinical case data arranged in a mapping form generated an active search and an exercise in self-learning in oncology. Despite the challenges inherent in the use of CM, the results suggested an increase in autonomy and clinical reasoning in nursing practice. Another study in nursing education, conducted by Kaddoura et al. [47], explored how students perceived the effect of a CM on the development of clinical judgment skills. The students created CMs, which were later evaluated both by them and the clinical instructors. Students also completed a clinical evaluation questionnaire at the end of the course. A descriptive data analysis was performed after the course was completed. The findings revealed that the use of CM provided an interactive way to foster clinical judgment skills in nursing students. In a similar vein, Hsu et al. [48] showed how outcome-based CM can be used as an efficient educational method that encourages nursing students to take a bio-psycho-social approach to medicine, which might ultimately lead to improved results.

### Concept mapping and self-regulation of learning

In recent years, researchers have called on higher-education teachers not only to provide students with opportunities to construct concepts but also to utilize it to foster their ability to self-regulate their learning processes [14–16]. However, while CMs have been shown to be effective tools for facilitating students’ critical thinking, the benefit of CMs in facilitating their self-regulation abilities in face-to-face or online courses has been underexplored [13].

Most of the research investigating the relationship between CM and SRL skills has considered self-regulation as an outcome variable. As indicated by Novak [49], when students gained skill and experience in constructing CMs, they began to report that they were learning how to learn. For example, Chularut and DeBacker [14] maintain that CM is designed to support students in self-regulating their learning. Strategies such as CM can help students attend to tasks, focus on important features, organize material, and maintain a productive psychological climate for learning. While working on CMs, students have very concrete evidence of whether and how well their CM is developing in the direction of their goal. Upon self-observation, they evaluate their CMs with regard to the standards and/or the goals they hold for themselves. Following self-observation and self-judgment, the students experience either satisfaction or dissatisfaction with regard to their progress or their completed map and may react by seeking further information or assistance. Satisfaction with their learning progress is likely to motivate students to use CM as a learning strategy in other settings.

Similarly, Naderifar [15] investigated the effects of CM on Iranian-born English as a Foreign Language (EFL) learners’ self-regulation. One group was given CMs, another group received a notebook, the last group served as the control group. The results of the analysis revealed that both CM and notebook keeping significantly enhanced SRL in vocabulary learning among the learners. Other researchers (e.g., Roy [16]) also revealed that students in an EFL context gained higher self-regulation as the result of CM strategy teaching. Though limited to EFL studies, these findings have implications for both pedagogy and research. These findings commonly indicate that CM increases readers’ autonomy, information comprehension, retention, and recall and additionally promotes uniquely individual performance.

Siebene et al. (2020) [9] investigated how concept mapping may support the quality of reflections made by undergraduate medical students. Reflection is viewed as a self-regulatory skill. Learners who are encouraged to evaluate their work through reflection can develop individual strategies that support their learning [50]. To nurture medical students’ reflective writing skills, concept mapping was used by Sieben et al., 2020 [9] as a format for reflection, supporting students to freely shape their thoughts. Concept mapping was detected as a useful tool to teach learners the basics of effective reflection.

However, since CM activity requires high mental effort while making meaning, it is equally plausible to posit SRL as an independent variable. Lim et al. [51] suggest that those who possess weak SRL skills are probably less able to direct their own learning, and therefore may gain less benefit from CM strategy. Since learners process information differently, strategies such as CM might not work equally well for all learners [52].

Drawing on the motivational control aspect of SRL, several studies have shown how self-regulated students are more receptive to adopting CM strategies. For example, Schaal [53]argues that self-efficacy might moderate the application of a meta-cognitive technique like CM. Consequently, students who believe they are capable of using CMs are more likely to participate in this activity. Similarly, Sun and Chen [54] showed how the use of CMs significantly increased elementary school students’ self-efficacy when their initial self-efficacy was already high to begin with. They suggest that this type of teaching/learning strategy is more suitable for improving these students’ self-efficacy than that of students with initially low self-efficacy beliefs. For learners who believe in their ability to acquire knowledge, they suggest that teachers adopt dynamic CMs and integrate them in their educational materials and questions designed to promote learners’ thinking and enhance their learning achievement.

Other researchers, such as Nuuyoma and Fillipus (2020) [55] showed that CM strategies are challenging for students with poor self-regulated learning skills such as time management. The researchers used concept mapping to facilitate learning among nursing students in the human physiology course. Their qualitative study aimed to describe the students’ perceptions and experiences while using concept mapping as a learning tool. Focus group discussions were analyzed yielding four themes: concept mapping facilitates deep learning; concept mapping as a group activity; effects of concept mapping on students’ academic performance; and implications of concept mapping for learning resources. Students had positive experiences and perceptions of concept mapping as a learning tool, however, they felt that this tool is time consuming and necessitates many learning resources. Therefore, the authors concluded that students should be guided on time-management strategies, an important aspect of SRL, to encourage the adoption of CM strategies.

### This study

The literature review indicates that CMs are still underutilized in health education and that little is known about how higher education students’ SRL might be linked to the way in which they use CMs for effective problem-based online courses. To address this utilization gap, the overarching aims of this study were to design a PBL intervention (see description below) that employs CM with a designated decision-making process (CM for PBL) and to evaluate the set of connections between this intervention and the SRL of Management of Health Service Organizations program students. The intervention was piloted for three months (one semester) in one central Israeli academic college. The following research questions and hypotheses were checked:

*(Q1)* The first research question was: how might students’ SRL be related to their perception of CM for PBL in an online course? Based on a literature review [51], the level of SRL skills might be suggested as an independent variable. High self-regulated students were expected to be more receptive to adopting CM and to demonstrate a predilection to utilize this learning strategy, which offers ways to effectively demonstrate their solutions to an ill-structured problem compared to low self-regulated learners. Therefore, it was postulated that health management students’ SRL will be positively linked to their perceptions of CM for PBL in an online course (*H1*). This hypothesis was checked following Phase 1 of the intervention.

*(Q2)* The second research question was: how will CM for PBL impact students’ SRL in an online course? Given the positive effect on SRL attributed to CM [15], it was hypothesized that the activity would enhance students’ SRL (*H2*). An additional hypothesis was that students’ SRL will be found higher after the intervention (Phase 2) than before the intervention (following Phase 1) (*H3*).

Additional variables, such as gender and age, were addressed to examine and control for their potential effect on the research constructs.

## Method

### Participants

Data for the analysis were gathered from 100 Israeli undergraduate students of a Management of Health Service Organizations program (covering patient-doctor relations, quality of service in the healthcare system, and ethics and patient rights). The program instructs students regarding the fundamentals in fields such as marketing, finance, organizational behavior, communications, legal issues and strategies. The students were enrolled in a 3rd-year course entitled ‘Assimilation of service quality in health systems’. The mean age of the participants was 25.40 years (SD = 6.76), and 86% were females. The distribution regarding ethnicity was: 51% Jewish students; 49% Arab (Muslim and Christian) minority students.

Data were gathered twice, after each phase of the intervention, as described in the following section. Prior to obtaining participants’ consent (informed consent was obtained from all participants involved in the study), it was explained that the questionnaires were anonymous and that it was acceptable should they choose to return a partially completed questionnaire. Finally, participants were assured that no specific identifying information would be processed. The study was preauthorized by the college’s Ethics Committee and in accordance with the relevant guidelines and regulation.

### The intervention

Grounded in a number of theories identified with the constructivist approach to learning such as cognitive schemes, moral development theory, dilemma discussion, moral reasoning schemes, radical theory, and social-cultural constructivist theories, the approach to learning known as Values and Knowledge Education (VaKE) [56] was designed and piloted in this study. VaKE is deemed a useful teaching tool that combines morality and values-centered education with knowledge education, with an emphasis on social behavior and the development of critical thinking in a PBL environment. In line with VaKE, the students were presented with the following dilemma, relevant to their course content.

#### The accreditation dilemma

Accreditation is a process by which an independent external entity evaluates organizations that provide healthcare services to determine whether they meet the set of standards and demands that are aimed at improving the quality of treatment. To date, there has been only scant research that supports the benefits of the accreditation process, although various studies do exist that point to the contribution of the accreditation process to promoting quality in healthcare organizations in general, and in hospitals in particular. Yet the process has numerous disadvantages such as a lack of manpower and inadequate funding. The increasingly strict quality control inspections, which have existed in Israel for more than a decade, became an obligatory condition for renewing hospital licenses in July of 2015. There is no doubt that the accreditation has made a huge contribution to hospitals. The competitive spirit that arose and the desire to obtain an international stamp of approval spurred hospitals to implement structural and cultural changes. It additionally served as an incentive for implementing basic work processes, most of which occurred in the fields of standardization, order, organization, and management. In practice, accreditation compelled the hospitals to undergo an essential reorganization, from procuring resuscitation equipment and defining jobs to implementing rules and regulations. Additional parameters were added to accreditation from year to year, and the cumulative burden forced the nurses to demand that it be eliminated altogether. They claimed that it hindered their work to the point at which they could no longer function properly. There is no debate in the profession as to the necessity of quality control and improving medical processes and procedures in the hospitals. The report of quality indexes of the Ministry of Health provides strong evidence of the positive impact of accreditation. However, the main problem stems from the cost involved in inspections, particularly those that require extra manpower. When the Ministry of Health established accreditation as an obligatory standard, the hospitals did not receive any supplementary budgets and were forced to pay most of the expenditure from their own pockets. Continuing the existing situation undoubtedly poses a challenging dilemma for the Ministry of Health and the hospitals with regard to the hospitals’ obligation to implement this process and ultimately attain approval.

The students in this research were asked to argue for or against the implementation of the accreditation process within hospitals. The task had two phases. In Phase 1, the participants were asked to detail five arguments to establish their decision by using a CM. Group work was allowed, although individual work was preferable. In Phase 2, relying on the materials taught in their courses, the students were asked to obtain the necessary supporting information to substantiate their arguments and to associate the ethical values at stake with at least two of the arguments they had provided. Finally, the participants were instructed to specify and explain the differences or similarities between their respective arguments. *Mindomo*, a popular platform for designing CMs, was utilized. To facilitate assessment of their maps, well-established criteria were provided to the students, as illustrated in Table 1. This assessment tool was adapted from Panadero et al. [57] to address the current study’s goals.

**Table 1:** Rubric for assessing the concept map (CM)

Criteria / Score	4	3	2	1
**Arguments and supporting information**	All five arguments and five justifications with supporting items of information are included.	Three-four arguments and justifications with supporting items of information are included.	One or two arguments and justifications with supporting items of information are included.	Arguments and justifications with supporting items of information are incomplete and/or incorrect.
**Hierarchy**	The organization is complete and correct. The supporting information corroborates the arguments.	The organization is correct but incomplete. Most of the supporting information corroborates the arguments.	The organization is correct but incomplete. Most of the supporting information does not corroborate the arguments.	The organization is incomplete and/or incorrect.
**Relationships among arguments / supporting information**	Relationships were specified and well-explained. Links to ethical values were added and explained.	Relationships were partly specified but explained. Links to ethical values were partly added but explained.	Relationships were partly specified but not explained. Links to ethical values were partly added but not explained.	Relationships were partly or not specified and poorly/not explained. Links to ethical values were incorrect or missing.
**Simplicity and easiness of understanding**	The design is simple and easy to understand.	Some relationships are difficult to understand.	There is an excessive number of connections.	Neither the relationships nor the hierarchy are understandable.

**Fig. 1:**
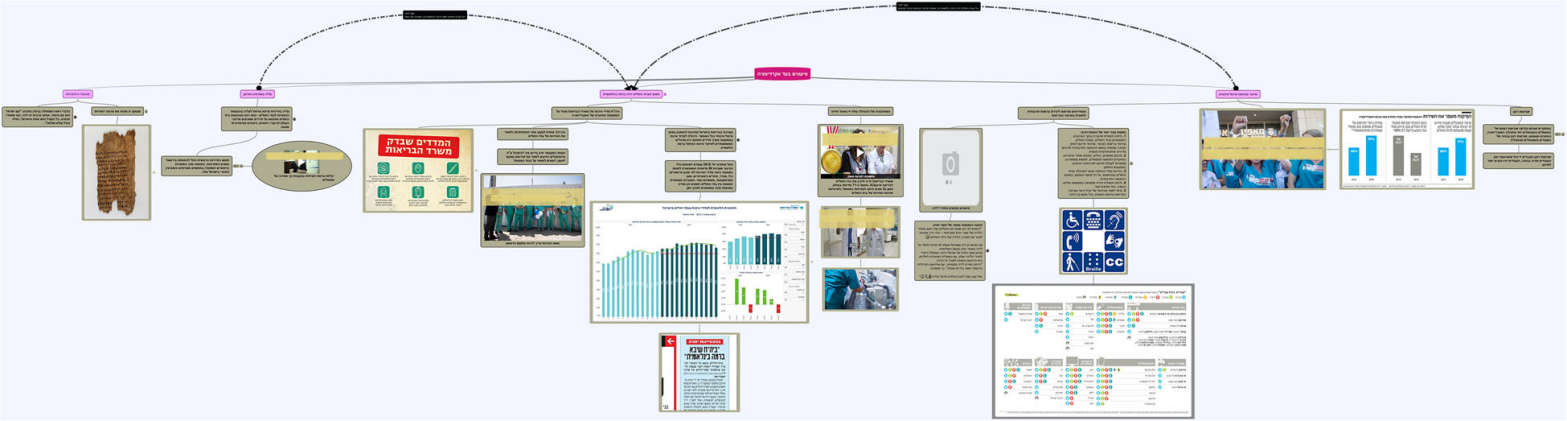
A concept map detailing the arguments in favor of implementing a hospital accreditation process

Figure 1 displays a map that illustrates the arguments in favor of assimilating an accreditation process in hospitals together with their textual support. An example of a basic argument is improving safety and risk management; for example, an increase in client satisfaction, a decrease in the number of infections/contaminations, and fewer legal claims. These are accompanied by supporting evidence using a variety of formats such as films, figures, tables, excerpts from academic articles, and articles from the local press. The full sources are added as weblinks next to each justification. The connections between the various arguments are marked on the map and accompanied by a written explanation. In the example shown here, formulating safety improvements and risk management will strengthen the argument that deals with improving the hospital’s reputation. The ethical values that were attributed to each argument are also explained; for example, preserving the value of human life by preventing hospital-acquired infection, or preserving human dignity by protecting medical confidentiality and, above all, the health of the patients.

### Measurements

#### Student characteristics

Data were gathered using a questionnaire aimed at ascertaining the student’s gender, age, and ethnicity.

#### Concept mapping for problem-based learning scale (CM for PBL)

A 12-item questionnaire was designed based upon the theoretical framework of CM and VaKE and the authors’ expertise in the field of moral decision-making process in PBL ([58];see Table 2). The questionnaire was aimed at assessing students’ perceptions of the effectiveness of using CMs in the decision-making process required in VaKE. The participants were asked to indicate their level of agreement with each of the statements shown in Table 2. The items were scored on a six-point Likert scale ranging from 1 = *strongly disagree* to 6 = *strongly agree*. The questionnaire was given twice, following the first phase (α = .97), and following the second phase (α = .97).

**Table 2:** Concept Mapping for PBL scale item description

1	Concept mapping helped me learn about the topic.
2	Concept mapping helped me identify the interrelationships among arguments.
3	Concept mapping helped me specify interrelationships among arguments.
4	Concept mapping stimulated me to learn and think independently.
5	Concept mapping helped me to reduce barriers when dealing with decision-making.
6	Concept mapping enhanced my interest in decision-making.
7	I think concept mapping can be easily used in other decision-making discussions.
8	I will consider using concept mapping in other complex decision-making processes.
9	I will consider using concept mapping to make decisions in the future.
10	I was satisfied using concept mapping in making decisions.
11	I liked using concept mapping to assist me in making decisions.
12	I enjoyed using concept mapping during the decision-making process.

#### The online self-regulated learning questionnaire (OSLQ)

This 24-item scale was developed by Barnard et al. [13] from an 86-item pool and then examined for its internal consistency and exploratory factor analysis results for data collected. Higher scores on this scale indicate better self-regulation in online learning by students. The items were scored on a six-point Likert scale ranging from 1 = *strongly disagree* to 6 = *strongly agree*. The OSLQ consists of six subscale constructs including: environment structuring (‘I find a comfortable place to study’); goal setting (‘I set standards for my assignments in online courses’); time management (‘I allocate extra studying time for my online courses because I know they are time-demanding’); help-seeking (‘I find someone who is knowledgeable in course content so that I can consult with him or her when I need help’); task strategies (‘I read aloud instructional materials posted online to cope with distractions’); and self-evaluation (‘I communicate with my classmates to find out how I am doing in my online classes’).

A principal axis factoring analysis followed by a varimax rotation was used to corroborate the stability of the scale structure (eigenvalue > 1.00; item loadings > .30). The analysis solution accounted for 58.45% of the variance and yielded five categories: Goal setting (items: 1–5 α = .82); Environment structuring (items: 6–8 α = .81); Task strategies (items: 12, 13 α = .87); Time management (items: 14–16 α = .87); and Peer support (items: 17, 19, 23, 24 α = .80). The last category included items from two original factors, self-evaluation and help seeking.

This scale was given to the participants twice, after each phase (1 and 2). Structural validity was established by using the same procedure. The analysis solution accounted for 59.46% of the variance. Five factors were found, with sufficient internal reliability results ranging from α = 0.66 for the 2-item Task strategies factor to α = 0.87 for the 3-item Time management factor.

### Data analysis

**Fig. 2:**
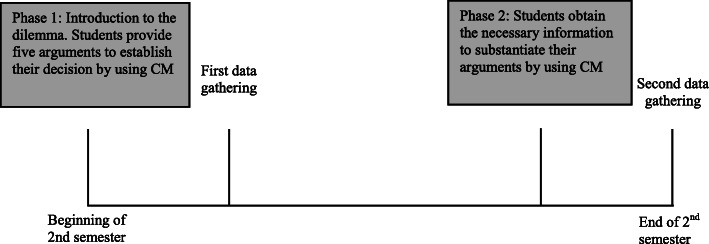
Research design

Data were analyzed using Partial Least Squares - Structural Equation Modeling (PLS-SEM) [59],which is recommended to be applied provided that the primary objective of employing structural equation modeling is the prediction of target constructs. SmartPLS 3 software was used. Figure 2 illustrates the research design.

### Findings

#### First hypothesis

**Fig. 3:**
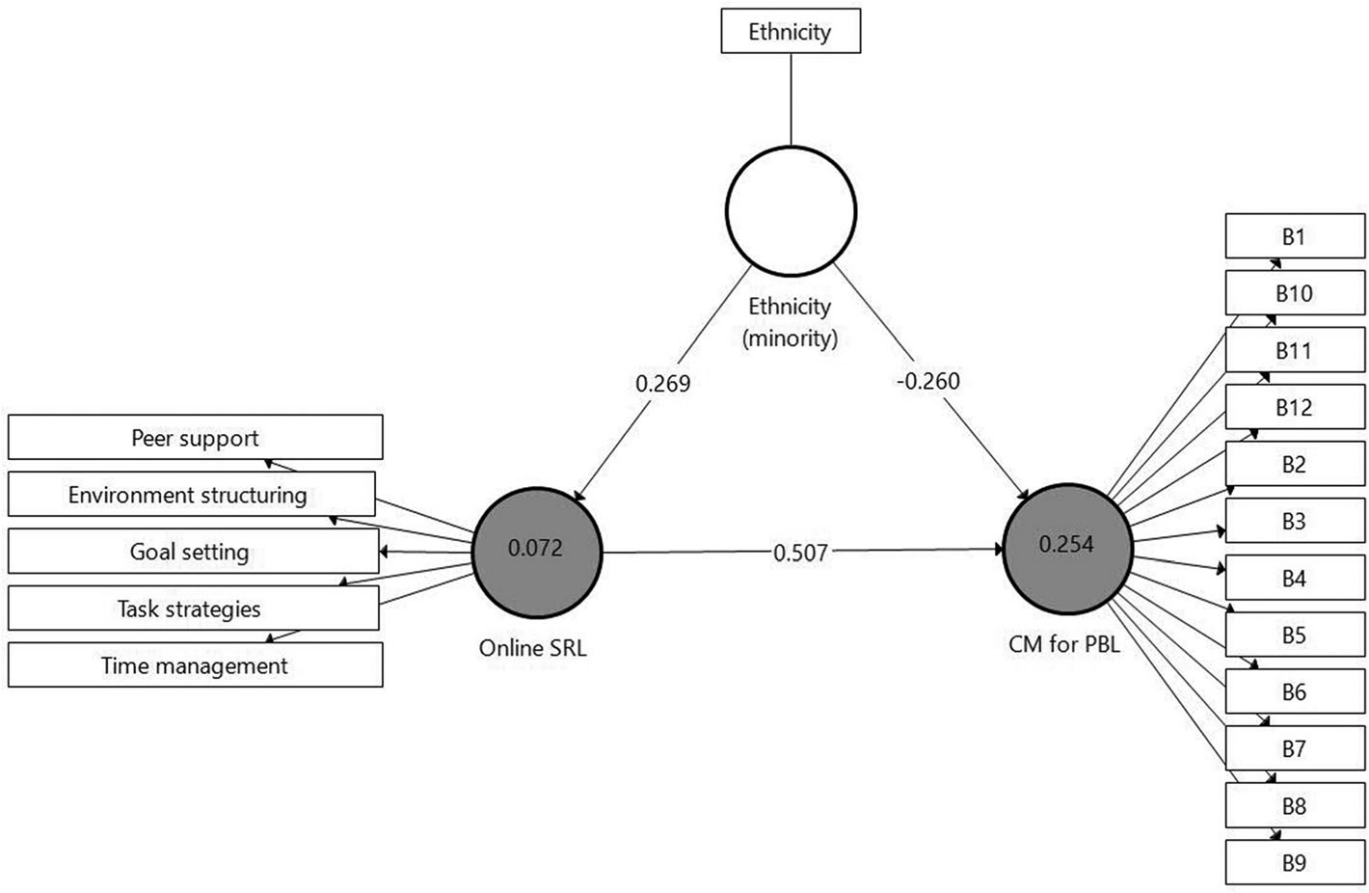
Model 1. Analysis results of the examination of *H1* by SmartPLS

In *H1* it was postulated that students’ SRL will be positively linked to their perceptions of CM for PBL in an online learning environment. To test this hypothesis, Model 1 (Fig. 3) was designed using data gathered in Phase 1. The model includes two main latent constructs: on the left, the Online SRL factor with its five sub-factors and, on the right, the perception of CM for PBL factor, accompanied by its 12 indicators. The indicators are the directly measured proxy variables, represented as rectangles. Relationships between the constructs as well as between the constructs and their assigned indicators are shown as arrows. In PLS-SEM, single-headed arrows, as shown between the constructs, are considered predictive relationships and, with strong theoretical support, can be construed as causal relationships. Paths were specified from Online SRL the CM for PBL-dependent factor. The PLS-SEM analysis used a path-weighting scheme and a mean value replacement for missing values. It should be noted that based on a previously conducted analysis, background variables were also entered into the model to control their effect on the latent variables (age, gender, and ethnicity). The Ethnicity variable (*majority* = 1, *minority* = 2) was found significantly connected to the model’s constructs and, therefore, was included in the model.

To test the direct effects, we ran the bootstrap routine. Bootstrapping makes no assumptions about the shape of the variables’ distribution or the sampling distribution and can be applied to small sample sizes [59]. As can be learned from Model 1, the Online SRL was found positively connected to the CM for PBL factor (β = .507 *p* < .001). Regarding cultural differences, minority students were more reluctant to embrace CM for PBL (β = −.260 *p* < .001) and had a high level of Online SRL compared to the majority students (β = .269 *p* < .05). Therefore, the first hypothesis was confirmed.

#### Evaluation of model 1

The coefficient of determination (*R*^*2*^) value was examined, whereby *R*^*2*^ values of 0.75, 0.50, or 0.25 for endogenous latent variables can be respectively described as substantial, moderate, or weak [59]. *R*^*2*^ for CM for PBL was found weak (0.254). In addition to measuring the *R*^*2*^ values, the change in the *R*^*2*^ value when omitting a specified exogenous construct from the model was used to evaluate its impact on the endogenous constructs. This measure is referred to as the *f*^*2*^ effect size whereby values of 0.02, 0.15, and 0.35, respectively, represent small, medium, and large effects [60]. *f*^*2*^ effect size result was 0.320 for Online SRL – CM for PBL, while *f*^*2*^ effect size results for the background variable were very low (0.078 and 0.084). Finally, the blindfolding procedure was used to assess the predictive relevance (*Q*^*2*^) of the path model. Values larger than 0 suggest that the model has predictive relevance for a certain endogenous construct [59]. The *Q*^*2*^ value of CM for PBL in the present study was 0.183.

**Fig. 4:**
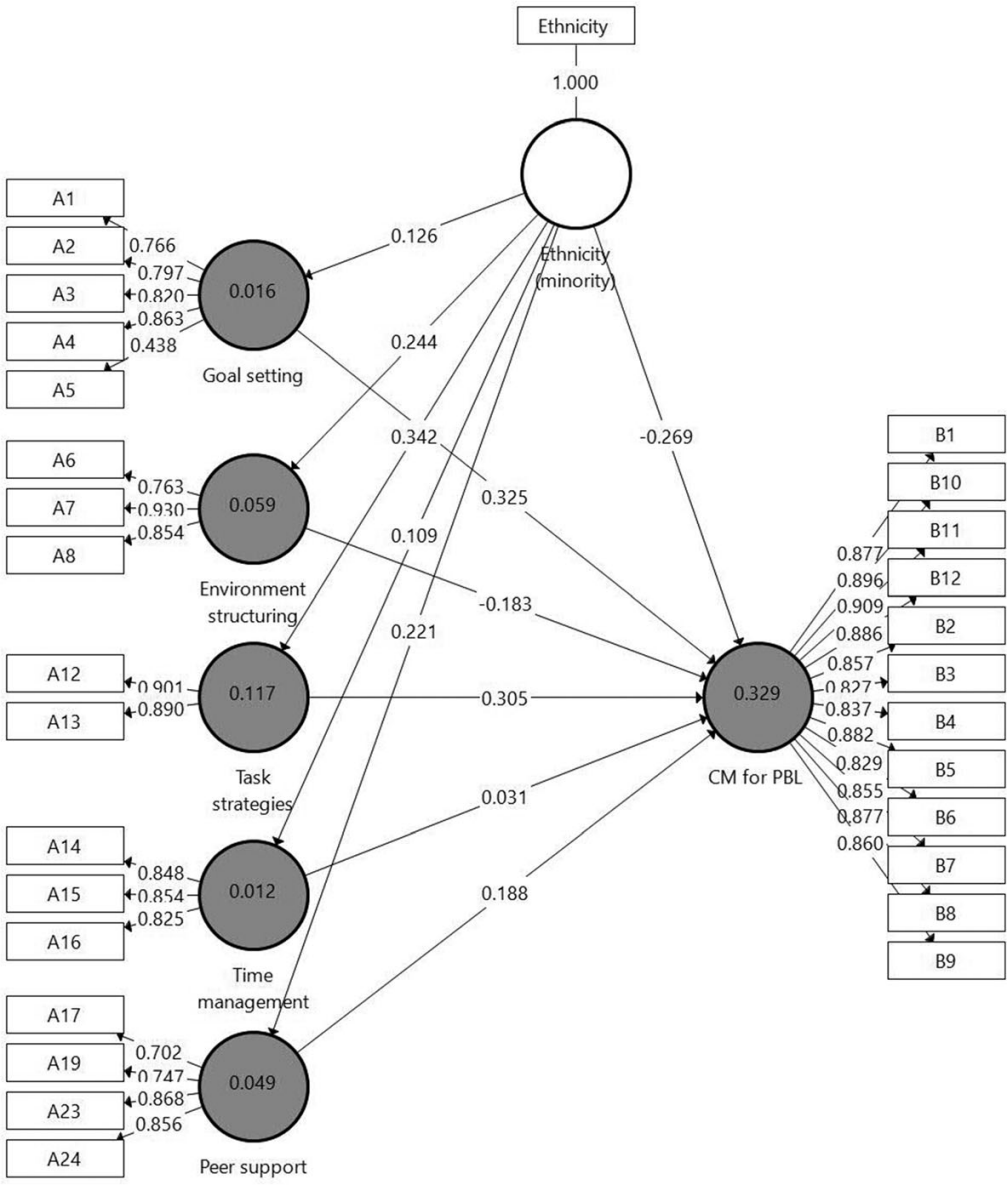
Model 2. Further analysis results of the examination of *H1* by SmartPLS

To evaluate the value of the Online SRL sub-factors in predicting students’ perceptions of the effectiveness of using CM in the decision-making process required in the VaKE, Model 2 (Fig. 4) was designed. The model includes the same latent constructs as those in Model 1; however, this time the Online SRL sub-factors were entered into the model as five independent variables. As shown in Model 2, only two sub-factors were found significantly connected to CM for PBL: Goal setting (β = .325 *p* < .01) and Task strategies (β = .305 *p* < .01). Non-significant connections were shown between other Online SRL sub-factors and the dependent variable.

#### Evaluation of model 2

The model evaluation included several steps. First, collinearity was examined by the Variance Inflation Factor (VIF) values of all sets of predictive constructs in the structural model. The results showed that the VIF values of all combinations of endogenous and exogenous constructs are below the threshold of 5 [59], ranging from 1.00 to 2.19. Therefore, collinearity among the predictive constructs is not a critical issue in this structural model. *R*^*2*^ for CM for PBL was found to be moderate (0.329). According to the *f*^*2*^ effect size results, a medium effect (0.071) was exerted by Goal setting, and Task strategies (0.071) on CM for PBL whereas smaller effects were shown for the other independent sub-factors. Finally, the *Q*^*2*^ value for CM for PBL was 0.238.

### Second hypothesis

**Fig. 5:**
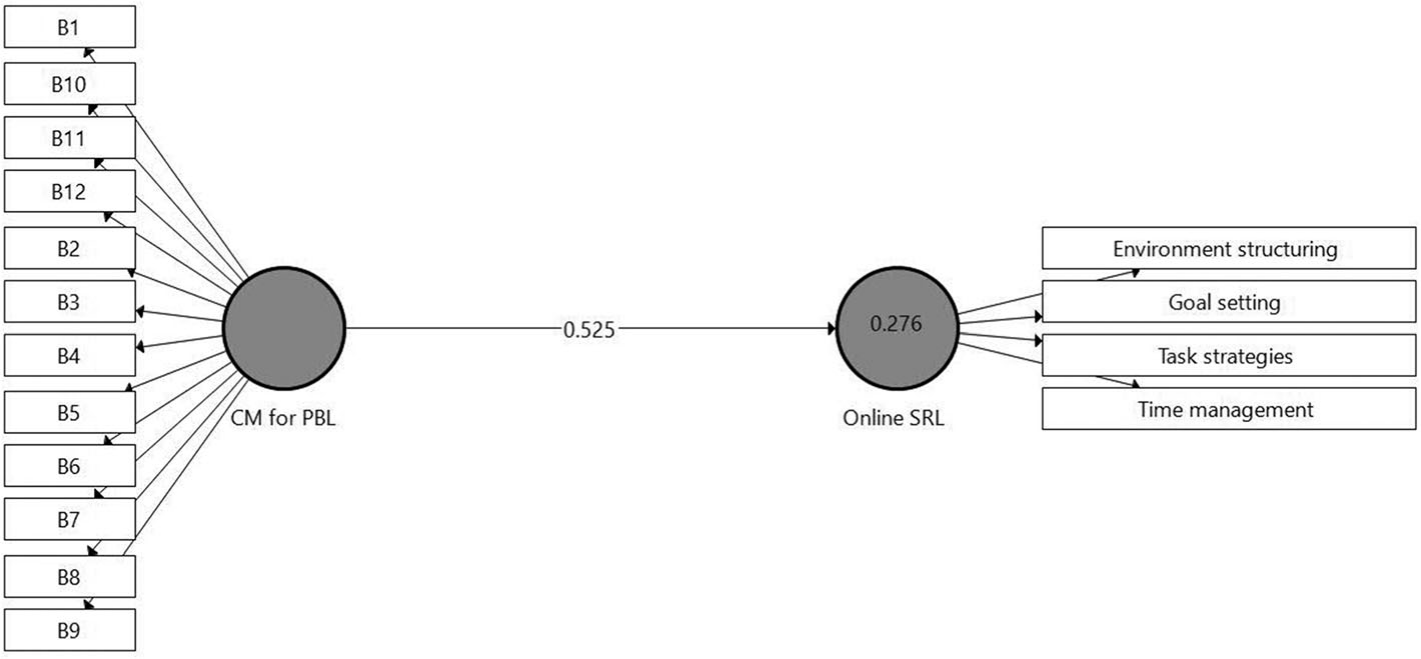
Model 3. Analysis results of the examination of *H2* by SmartPLS

In *H2* it was expected that the activity of CM for PBL will enhance students’ SRL (*H2*). To check this hypothesis, Model 3 (Fig. 5) was designed to include data gathered in Phase 2 (following the intervention). As can be seen in Fig. 5, the model includes the CM for PBL independent factor and the Online SRL dependent factor with four sub-factors (the Peer support sub-factor was omitted from the analysis due to a low loading result < 0.40; see Hair et al. [59]). Background variables, including Ethnicity were found non-significantly connected to the model’s constructs and were therefore excluded from the model. According to the bootstrap routine, the CM for PBL factor positively contributed to students’ Online SRL (β = .525 *p* < .001). Regarding model evaluation results, *R*^*2*^ for Online SRL was found to be moderate (0.276). The *Q*^*2*^ value for Online SRL was 0.162. Therefore, the second hypothesis was confirmed.

**Fig. 6:**
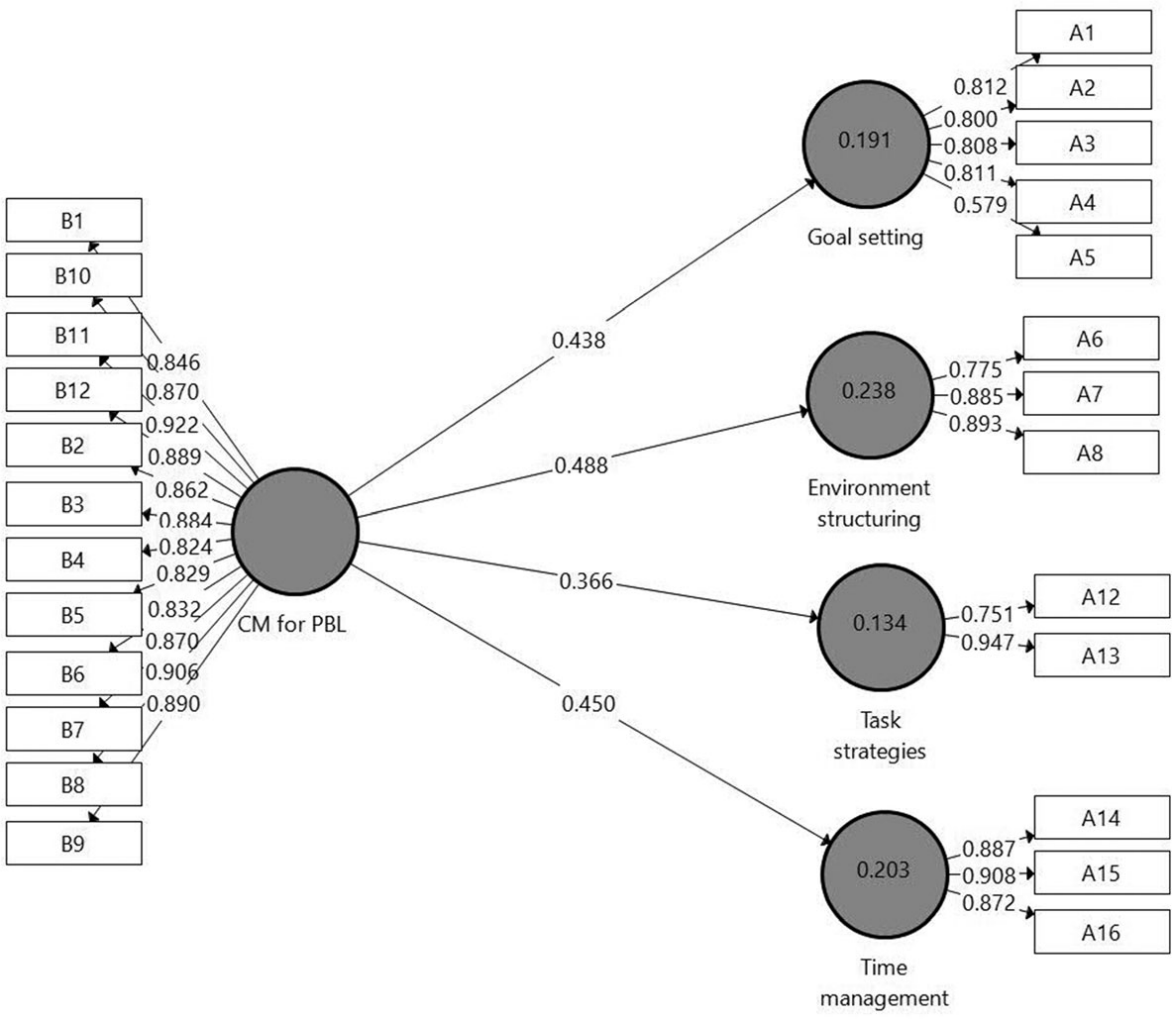
Model 4. Further analysis results of the examination of *H2* by SmartPLS

To assess the contribution of CM for PBL to Online SRL sub-factors, Model 4 (Fig. 6) was designed. The model includes the same latent constructs as in Model 3; however, this time the Online SRL sub-factors were entered into the model as four independent variables. As shown in Fig. 6, CM for PBL was found significantly linked to all of the Online SRL sub-factors: Goal setting (β = .438 *p* < .001), Environment structuring (β = .488 *p* < .001), Task strategies (β = .366 *p* < .001), and Time management (β = .450 *p* < .001). Regarding model evaluation results, a collinearity examination yielded sufficient results (i.e., VIF values were equal to 1.00). As indicated in Model 4, the highest *R*^*2*^ result was indicated for Environment structuring (*R*^*2*^ *=* 0.238), and the lowest for Task strategies (*R*^*2*^ *=* 0.134). The *Q*^*2*^ values ranged from 0.087 to 0.150.

### Third hypothesis

Finally, an additional analysis was conducted to detect possible contribution of CM for PBL to Online SRL sub-factors. A repeated-measures analysis yielded a significant result (*F*_(188, 6)_ = 3.656, *p* < .01, η^2^ = .104) in students’ Online SRL between Phase 1 and Phase 2. SRL Time management sub-factor (*F*_(188, 1)_ = 4.811, *p* < .05, η^2^ = .024), was found significantly higher after the intervention (Phase 2) relative to its beginning (Phase 1). Table 3 presents the mean scores, *SD*, *F* values, and Eta-squared statistics (η^2^) of Online SRL (Phase 1 and 2).

**Table 3:** Mean scores, *SD*, *F* values, and Eta-squared *statistics* (η^2^) of Phase 1 and 2

Factors	Phase 1		Phase 2			
	*M*	*SD*	*M*	*SD*	*F*	η^2^
Online SRL						
Goal setting	4.54	0.90	4.59	0.88	0.175	.676
Environment structuring	4.58	1.04	4.31	1.06	3.148	.078
Task strategies	3.37	1.34	3.71	1.31	3.310	.070
Time management	4.02	1.18	4.38	1.14	4.811*	.029
Communication with classmates	3.60	1.19	3.63	1.23	0.025	.874

*p* < .05 *

## Discussion

This study mainly shows how students with different SRL in online courses might tend to differently utilize a learning strategy that offers ways to effectively demonstrate their solutions to an ill-structured problem using CM. Next, it illustrates the effectiveness of the CM for PBL strategy for students’ SRL in an online course. As postulated, at the beginning of the process, students’ SRL was found positively linked to the CM for PBL strategy. However, only two SRL sub-factors exerted a positive effect on the learning strategy: Goal setting and Task strategies. It may be inferred that students who were accustomed to setting standards for their assignments in online courses, set short-term goals as well as long-term goals, kept a high standard for their learning in online courses and for the quality of their work, and undertook extra problems in their online courses to master the content, were found more receptive to adopting CM for PBL online course and demonstrated a predilection to utilize this learning strategy. This can be corroborated by previous researchers who claim that self-regulated learners are individuals who are already assessing their own performance to varying degrees, while generating their own self-feedback actively and consciously controlling their own learning from cognitive, affective, motivational, and behavioral points of view [61]. In view of its findings, the present study adds to the corpus of knowledge by pointing to a specific learning activity – CM –revealed by self-regulated learners as being an important and useful tool that helped them take control of their learning and tackle the PBL assignment.

Another interesting finding was shown in the second analysis with data gathered following the intervention (Phase 2). The contribution of CM for PBL to Online SRL sub-factors was found to be significant. The analysis showed that the CM for PBL learning strategy increased the levels of four Online SRL sub-factors: Goal setting, Task strategies, Environment structuring, and Time management. The last two factors failed to predict the perception of the learning strategy at the beginning of the intervention. These findings show that the CM for PBL learning strategy motivated students to choose a quiet study venue to avoid distractions and study optimally for the online course, to allocate extra studying time for their task, and distribute their studying time evenly throughout the course.

Finally, increased levels of Time management were found upon completion of the intervention, as compared to its starting point. Time management represents a strategic process that promotes the accomplishment of important goals and successes within personal, professional, and academic contexts [62]. Academic time management describes students’ efforts to allocate the use of their time purposefully in order to achieve important educational goals within a given period of time [63]. Time management is characterized as a multidimensional process through which students deliberately control the way they engage in academic work; hence it has been portrayed as a manifestation of students’ regulation of their overt behavior [64]. It may be inferred that the use of such a self-regulatory strategy involves a level of engagement that is often more demanding for students in terms of time and effort than their normal level of engagement [65]. In order for them to invest the extra time and effort, the learning environment must be designed in a way that motivates them to allocate time to successfully accomplish the task and encourages and scaffolds students’ effective use of SRL strategies. Based on this study, the CM for PBL strategy can be suggested as an effective online strategy that motivates students to regulate their behavior.

It is thus important to note that our results might be connected to the way the activity was designed in this research. The students were given predefined criteria that allowed them to self-assess their maps. It is plausible to assume that because the activity included this assessment tool, aimed at helping students improve their abilities through progressive goal setting, the students found it valuable and used it as scaffolding to enhance their learning skills. Arguably, strategies of self-evaluation and monitoring are considered vital for students‘SRL [66]. Such strategies, which increase student’s capacity to judge their own performance and results, are considered crucial for cultivating self-regulation [67].

### Limitations and directions for future studies

The present work features several limitations that merit a mention and opens avenues for future research. First, this study proposes a new measurement that captures students’ CM for PBL process. The results seem to confirm its validity with SRL in online activities used as a criterion variable. However, future studies should examine how it relates to scores from an instrument designed to assess a construct it would theoretically be related to, for example, the self-determination theory of motivation that aims to explain individuals’ goal-directed behavior [68]. Second, gathering qualitative data alongside quantitative measures might be a useful way of assessing and understanding the effectiveness of CM. Combining CM with a reflective journal might be a useful way to gather qualitative data [69] and increase students’ SRL [70]. Third, although entered into the model merely to control its effect on the research constructs, the Ethnicity factor should be further addressed in future studies. It seems that minority students were initially more reluctant to embrace CM for PBL despite their higher level of Online SRL, whereas this discrepancy was resolved by the end of the activity. CM facilitated the undertaking of suitable actions to offset the diversity represented by immigrant or minority students [71]; hence, further research should be done to investigate how CM can be used as an efficient instructional scaffold to support diverse students’ learning processes. Finally, this study was conducted in a single course and was limited to Management of Health Service Organizations program students in Israel; therefore, the results cannot necessarily be generalized to students of other countries and study tracks. Cross-cultural validation of the results is needed to substantiate the findings as well as to validate the factorial structure and the relationship among factors.

## Conclusions and implications

This study set out to fill the lacuna of information and practical designated training in the fields of medicine and medical management with respect to the wide variety of personal and interpersonal qualifications that are necessary for adaptation to the twenty-first century and its various challenges. The perception that the objective of academia and institutions for medical education is to remain relevant to the demands of the twenty-first century calls for them to adapt themselves to the changing needs of the labor market and the health system. Consequently, their actions and learning must be adapted to the needs of the profession in the present and the future and to the everchanging challenges which are yet unknown. As part of this process, we must place an emphasis on the students in the system and train them by developing the necessary qualifications in early stages of their studies in medical schools, nursing schools, and medical management schools, as well as in the stages of advanced learning and professional training.

This study presents the development of SRL among Management of Health Service Organizations program students in the context of online PBL and the use of dilemmas and constructing a CM using designated programs. Combining these teaching and learning tools together with the use of advanced technology in an online course that encourages active learning enables the development and acquisition of abilities of self-directed learning among students in the medical and health management professions. This constitutes an additional step towards adapting the health system and its practitioners to the demands and needs of the twenty-first century.

Drawing on the results of the current study, it can be concluded that although the effects of CM interventions might vary with different ability learners, CM for PBL learning strategy can further enhance students’ SRL. This study shows that for a CM-based intervention to be effective in PBL online environments, teachers need to recognize and account for different types of learners and adapt their curricula and learning environment to encourage and scaffold students’ effective use of SRL strategies. For example, they can help straggling students create study schedules, thereby guiding the learning process towards goals that emphasize the successful acquisition of skills and knowledge. Low self-regulated learners might fail to see the advantages of CM in problem-solving activities. Well-defined assessment criteria that are shared in advance with the students may motivate them to invest adequate time and effort in the task. The students with insufficient SRL skills might need to be further informed of the benefits of CM for their online PBL activities.

Although still preliminary, these findings point to an exciting new venue for further research, the findings of which are likely to have an impact on how CM could be used in a PBL online setting that supports SRL. However, given the paucity of literature that systematically explores CM’s practical applications and their relation to learning, motivation, and engagement in health education, additional studies are needed to corroborate our findings. Such future efforts might increase the potential of implementing strategies for assessment that promote lifelong learning skills.

## Data Availability

Data are available upon request from Prof. Dorit Alt, email: doritalt@014.net.il Data are available also Mendeley open data repository: https://data.mendeley.com/datasets/ngy6mprc6r/draft?a=1dacd25a-91d2-47f9-83a6-6b5801683807 Other materials can be provided by the authors on request.

## References

[1] Jungwirth D, Haluza D (2019). Information and communication technology and the future of healthcare: results of a multi-scenario Delphi survey. Health Informat J.

[2] Naamati Schneider L. Strategic management as adaptation to changes in the ecosystems of public hospitals in Israel. Israel J Health Policy Res. 2020:9:65. 10.1186/s13584-020-00424-y PMC7685193 33234166

[3] van Houten-Schat MA, Berkhout JJ, van Dijk N, Endedijk MD, Jaarsma ADC, Diemers AD (2018). Self-regulated learning in the clinical context: a systematic review. Med Educ.

[4] Berkhout JJ, Helmich E, Teunissen PW, van der Vleuten CPM, Jaarsma ADC (2018). Context matters when striving to promote active and lifelong learning in medical education. Med Educ.

[5] Wachter R, Wehrwein P (2015). A conversation with Robert Wachter, MD. Reality bytes: Medicine’s bumpy ride into the digital age. Manag Care.

[6] Bilik O, Kankaya EA, Deveci Z (2020). Effects of web-based concept mapping education on students’ concept mapping and critical thinking skills: a double blind, randomized, controlled study. Nurse Educ Today.

[7] Garwood JK, Ahmed AH, McComb SA (2018). The effect of concept maps on undergraduate nursing students’ critical thinking. Nurs Educ Perspect.

[8] Odreman HA, Clyens D (2020). Concept mapping during simulation debriefing to encourage active learning, critical thinking, and connections to clinical concepts. Nurs Educ Perspect.

[9] Sieben JM, Heeneman S, Verheggen MM, Driessen EW. Can concept mapping support the quality of reflections made by undergraduate medical students? A mixed method study. Med Teach. 2020:1–9. 10.1080/0142159X.2020.1834081. 10.1080/0142159X.2020.1834081 33280482

[10] Yue M, Zhang M, Zhang C, Jin C (2017). The effectiveness of concept mapping on development of critical thinking in nursing education: a systematic review and meta-analysis. Nurse Educ Today.

[11] Bransen D, Govaerts MJB, Sluijsmans DMA, Driessen EW (2020). Beyond the self: the role of co-regulation in medical students’ self-regulated learning. Med Educ.

[12] Lajoie SP, Zheng J, Li S, Jarrell A, Gube M. Examining the interplay of affect and self regulation in the context of clinical reasoning. Learn Instruct. 2019. 10.1016/j.learninstruc.2019.101219.

[13] Barnard L, Lan WY, Tob YM, Paton VO, Lai S-L (2009). Measuring self-regulation in online and blended learning environments. Internet High Educ.

[14] Chularut P, DeBacker TK (2004). The influence of concept mapping on achievement, self-regulation, and self-efficacy in students of English as a second language. Contemp Educ Psychol.

[15] Naderifar A (2018). The comparative effect of concept mapping and vocabulary notebook keeping on Iranian EFL learners’ self-regulation in vocabulary learning. Cogent Educ.

[16] Roy D (2011). Designing concept maps from procedural visuals: an innovative approach towards information processing in EFL context. Int J Arts Sci.

[17] Brydges R, Butler D (2012). A reflective analysis of medical education research on self-regulation in learning and practice. Med Educ.

[18] Lucieer SM, Jonker L, Visscher C, Rikers RMJP, Themmen APN (2015). Self-regulated learning and academic performance in medical education. Med Teach.

[19] Schunk DH, Zimmerman BJ (1994). Self-regulation of learning and performance: issues and educational applications.

[20] Zimmerman BJ (1986). Becoming a self-regulated learning: Which are the key subprocesses?. Contemp Educ Psychol.

[21] Zimmerman BJ, Martinez PM (1986). Development of a structured interview for assessing student use of self-regulated learning strategies. Am Educ Res J.

[22] Pintrich PR, Schunk DH (2002). Motivation in Education: Theory, Research and Applications.

[23] Novak JD, Gowin DB (1984). Learning how to learn.

[24] Kinchin IM (2014). Concept mapping as a learning tool in higher education: a critical analysis of recent reviews. J Cont High Educ.

[25] Novak J (1981). Applying learning psychology and philosophy to biology teaching. Am Biol Teach.

[26] Jennings D (2012). The use of concept maps for assessment.

[27] Croasdell DT, Freeman LA, Urbaczewski A (2003). Concept maps for teaching and assessment. Comm Assoc Inform Syst.

[28] Machado CT, Carvalho AA (2020). Concept mapping: benefits and challenges in higher education. J Cont High Educ..

[29] Conceicao SCO, Samuel A, Biniecki SMY. Using concept mapping as a tool for conducting research: An analysis of three approaches. Cogent Soc Sci. 2017;3(1). https://www.tandfonline.com/doi/full/10.1080/23311886.2017.1404753.

[30] Aydogan T, Ergun S (2016). A study to determine the contribution made by concept maps to a computer architecture and organization course. Eur J Contemp Educ.

[31] Bressington DT, Wong W-K, Lam KKC, Chien WT (2018). Concept mapping to promote meaningful learning, help relate theory to practice and improve learning self-efficacy in Asian mental health nursing students: a mixed-methods pilot study. Nurse Educ Today.

[32] Tsourela M, Paschaloudis D, Fragidis G, Giouvanakis A, Manos R (2015). Collaboration learning as a tool supporting value co-creation. Evaluating students learning through concept maps. Procd Soc and Behc.

[33] Lin Y-T, Chang C-H, Hou HT, Wu KC (2016). Exploring the effects of employing Google docs in collaborative concept mapping on achievement, concept representation, and attitudes. Interact Learn Environ.

[34] Wu S-Y, Chen SY, Hou H-T (2016). Exploring the interactive patterns of concept map-based online discussion: A sequential analysis of users’ operations, cognitive processing, and knowledge construction. Interact Learn Environ.

[35] Martin LG, Martin FA, Southworth E (2015). A critical review of concept mapping research literature: informing teaching and learning practices in GED preparation programs. New Hor Adult Educ and Hum Resour Dev.

[36] Greenberg RK, Wilner NA (2015). Teaching and educational notes: using concept maps to provide an integrative framework for teaching the cost or managerial accounting course. J Account Educ.

[37] Chen MRA, Hwang GJ (2020). Effects of a concept mapping-based flipped learning approach on EFL students’ English-speaking performance, critical thinking awareness and speaking anxiety. Brit J Educ Technol.

[38] Alfayoumi I (2019). The impact of combining concept-based learning and concept-mapping pedagogies on nursing students’ clinical reasoning abilities. Nurse Educ Today.

[39] Partnership for 21st Century Skills. ICT literacy maps: Social studies map; 2014. http://www.p21.org/images/stories/matrices/ICTmap_ss.pdf. Accessed September 29, 2020.

[40] Rosen Y, Mosharraf M. Evidence-centered concept map in computer-based assessment of critical thinking. In: Learning and Performance Assessment: Concepts, Methodologies, Tools, and Applications. USA: IGI Global; 2020. p. 656–82.

[41] Hwang G-J, Kuo FR, Chen NS, Ho HJ (2014). Effects of an integrated concept mapping and web-based problem-solving approach on students' learning achievements, perceptions and cognitive loads. Comput Educ.

[42] Hwang G-J, Zou D, Lin J (2020). Effects of a multi-level concept mapping-based question-posing approach on students’ ubiquitous learning performance and perceptions. Comput Educ.

[43] Latif RA, Mohamed R, Dahlan A, Mat Nor MZ (2016). Concept mapping as a teaching tool on critical thinking skills and academic performance of diploma nursing students. Educ Med J.

[44] Joshi U, Vyas S (2018). Assessment of perception and effectiveness of concept mapping in learning epidemiology. Indian J Community Med.

[45] Chan ZCY (2017). A qualitative study on using concept maps in problem-based learning. Nurse Educ Pract.

[46] Baig M, Tariq S, Rehman R, Ali S, Gazzaz ZJ (2016). Concept mapping improves academic performance in problem solving questions in biochemistry subject. Pak J Med Sci.

[47] Kaddoura M, VanDyke O, Cheng B, Shea-Foisy K (2016). Impact of concept mapping on the development of clinical judgment skills in nursing students. Teach Learn Nurs.

[48] Hsu LL, Pan HC, Hsieh SI (2015). Randomized comparison between objective-based lectures and outcome-based concept mapping for teaching neurological care to nursing students. Nurse Educ Today.

[49] Novak JD (1998). Learning, creating, and using knowledge: concept maps as facilitative tools in schools and corporations.

[50] Oates S (2019). The importance of autonomous, self-regulated learning in primary initial teacher training. Front Educ.

[51] Lim KY, Lee HW, Grabowski B (2009). Does concept-mapping strategy work for everyone? The levels of generativity and learners' self-regulated learning skills. Br J Educ Tech.

[52] Grabowski BL, Jonassen DH, Association for Educational Communications and Technology (2003). Generative learning contributions to the design of instruction and learning. Handbook of Research on Educational Communications and Technology. 2nd ed.

[53] Schaal F (2010). Cognitive and motivational effects of digital concept maps in pre-service science teacher training. Procd Soc and Behc..

[54] Sun JC-Y, Chen AY-Z (2016). Effects of integrating dynamic concept maps with interactive response system on elementary school students’ motivation and learning outcome: the case of anti-phishing education. Comput Educ.

[55] Nuuyoma V, Fillipus SK (2020). Nursing students’ perceptions and experiences of concept mapping as a learning tool in a human physiology course. Afr J Health Prof Educ.

[56] Patry J-L, Weinberger A, Weyringer S, Nussbaumer M. Combining values and knowledge education. In Irby BJ, Brown G, Lara-Alecio R, Jackson S, eds., and Robles-Piña RA, sect. Ed. The Handbook of Educational Theories*.* Charlotte, NC: Information Age Publishing; 2013: 565–579.

[57] Panadero E, Romero M, Strijbos JW (2013). The impact of a rubric and friendship on construct validity of peer assessment, perceived fairness and comfort, and performance. Stud Educ Eval.

[58] Alt D, Weinberger A, Heinrichs K, Naamati-Schneider L. Problem-based learning with digital concept mapping in Israeli and Austrian higher education settings: The association with students’ goal orientations and learning approaches. under review.

[59] Hair JF, Hult GTM, Ringle CM, Sarstedt M (2017). A primer on partial least squares structural equation modeling (PLS-SEM).

[60] Cohen J (1988). Statistical power analysis for the behavioral sciences.

[61] Boekaerts M, Pintrich P, Zeidner M (2005). Handbook of self-regulation.

[62] Wolters CA, Won S, Hussain M (2017). Examining the relations of time management and procrastination within a model of self-regulated learning. Metacogn Learn.

[63] Claessens BJC, van Eerde W, Rutte CG, Roe RA (2007). A review of the time management literature. Person Rev.

[64] Pintrich P, Zusho A, Perry RP, Smart JC (2007). Student motivation and self-regulated learning in the college classroom. The Scholarship of Teaching and Learning in Higher Education: An Evidence-Based Perspective.

[65] Pintrich PR (1999). The role of motivation in promoting and sustaining self-regulated learning. Int J Edus Res.

[66] Panadero E, Romero M (2014). To rubric or not to rubric? The effects of self-assessment on self-regulation, performance and self-efficacy. Assess Educ Princ Pol Pract.

[67] Zimmerman BJ, Moylan AR, Hacker DJ, Dunlosky J, Graesser AC (2009). Self-regulation: where metacognition and motivation intersect. Handbook of metacognition in education.

[68] Ryan RM, Deci EL (2000). Self-determination theory and the facilitation of intrinsic motivation, social development, and well-being. Am Psychol.

[69] Carrier A, Morin C (2014). Enabling students' self-regulation and teachers’ feedback in concept mapping. Med Educ.

[70] Alt D, Raichel N. Reflective journaling and metacognitive awareness: Insights from a longitudinal study in higher education. Reflective Practice. 2020;21:145–58.

[71] McCoy JD, Ketterlin-Geller LR (2004). Rethinking instructional delivery for diverse student populations: serving all learners with concept-based instruction. Interv Sch Clin.

